# Effects of Positive Airway Pressure on Cardiorespiratory Fitness in Patients with Concomitant Obstructive Sleep Apnea and Cardiovascular Disease

**DOI:** 10.3390/medicina60071029

**Published:** 2024-06-23

**Authors:** Kyusup Lee, Yu Jin Jung, Jung Sun Cho, Ji-Hoon Jung, Woojin Kwon, Jongbum Kwon

**Affiliations:** 1 Department of Cardiology, Daejeon St. Mary’s Hospital, College of Medicine, The Catholic University of Korea, Seoul 06591, Republic of Korea; ajobi7121@gmail.com (K.L.); tworugi@daum.net (J.S.C.); 2 Department of Neurology, Daejeon St. Mary’s Hospital, College of Medicine, The Catholic University of Korea, Seoul 06591, Republic of Korea; 3 Korea Institute of Toxicology, Daejeon 34114, Republic of Korea; jihoon.jung@kitox.re.kr; 4 Department of Anesthesiology and Pain Medicine, Konyang University Hospital, College of Medicine, Konyang University, Daejeon 35365, Republic of Korea; ktuttle81@naver.com; 5 Department of Thoracic and Cardiovascular Surgery, Daejeon St. Mary’s Hospital, College of Medicine, The Catholic University of Korea, Seoul 06591, Republic of Korea; jbkwon@catholic.ac.kr

**Keywords:** obstructive sleep apnea, positive airway pressure, cardiorespiratory fitness, cardiopulmonary exercise testing, ventilatory response

## Abstract

*Background and Objectives:* Obstructive sleep apnea (OSA) is common in cardiovascular disease (CVD), although positive airway pressure (PAP) treatment has not been demonstrated to improve the cardiovascular outcome. The objective of this study is to investigate the impact of adherence to PAP therapy on cardiopulmonary exercise testing (CPET) performance in patients with concomitant OSA and CVD. *Materials and Methods:* This preliminary study involved symptomatic OSA patients requiring PAP treatment who had CVD. All subjects underwent polysomnography, echocardiography, and CPET at baseline. After 6 to 12 months of PAP treatment, CPET performance was re-assessed. The changes in CPET parameters before and after PAP treatment were compared between patients who were adherent to PAP and patients who were not adherent to PAP. *Results:* A total of 16 OSA patients with an apnea–hypopnea index of 32.0 ± 23.4 were enrolled. Patients were classified into the adherent (*n* = 9) and non-adherent (*n* = 7) groups with regard to PAP adherence. After 6 to 12 months of PAP treatment, the PAP-adherent group showed a greater increase in peak VO2 than the PAP-non-adherent group, but the difference between the two groups was not significant (*p* = 0.581). The decrease in ventilatory equivalent for the carbon dioxide slope (VE/VCO2) was significantly greater in the PAP-adherent group compared to the PAP-non-adherent group (*p* = 0.030). *Conclusions:* Adherence to PAP therapy for OSA is associated with an improvement in the VE/VCO2 slope, as an index of the ventilatory response to exercise, in patients with CVD. Screening for sleep apnea in CVD patients may be warranted, and strategies to optimize adherence to PAP in these patients are beneficial. Further evidence is needed to elucidate whether CPET could be routinely used to monitor treatment responses of OSA to PAP therapy in patients with CVD.

## 1. Introduction

Obstructive sleep apnea (OSA) is characterized by recurrent episodes of complete or partial upper airway occlusion during sleep, resulting in intermittent hypoxia and hypercapnia, frequent arousals with sleep fragmentation, and excessive daytime sleepiness [1,2]. The prevalence of OSA is estimated to be from 9% to 38% in a population-based study utilizing an apnea–hypopnea index (AHI) cutoff of ≥5 events/h [3]. Common risk factors of OSA include male gender, increasing age, obesity, excess alcohol consumption, and menopause [4]. Moreover, goiter independently of thyroid function can be considered a risk factor for OSA [5]. With chronic exposure over time, OSA induces secondary sympathetic activation, oxidative stress, systemic inflammation, and endothelial dysfunction [6]. These alterations lead to an increased risk of cardiovascular disease (CVD), including hypertension, coronary disease, heart failure, ischemic stroke, or atrial fibrillation [7]. Cardiovascular disturbances have been considered one of the most serious complications of OSA [7]. 

Cardiorespiratory fitness (CRF), defined as the efficiency of the cardiovascular and respiratory systems in supplying oxygen to skeletal muscle during exercise, is an emerging concept for the management of numerous cardiopulmonary conditions [8]. Cardiopulmonary exercise testing (CPET) is a non-invasive technique, which provides an objective quantitative assessment of the integrated exercise responses involving the cardiovascular, pulmonary, hematopoietic, neuropsychological, and skeletal muscle systems involved in the oxygen transport chain [9]. CPET is a more sensitive method because it examines the subject not at rest, but under conditions where the body mobilizes its reserves during exercise. Given these characteristics, CPET to assess CRF is highly recommended in patients with an increased cardiovascular risk [8]. CRF, commonly reflected by the measurement of the maximum oxygen consumption (peak VO2) during CPET, is a reliable predictor of cardiovascular disease and mortality [8]. 

Despite the clear evidence of cardiovascular risk in OSA patients, previous studies that evaluated the cardiopulmonary response to exercise in these patients have shown conflicting results. Some studies have reported reduced maximal aerobic exercise capacity in patients with OSA compared to control subjects [10,11], while other studies have suggested that exercise capacity is preserved in OSA patients [12,13]. Recently, an individual patient data meta-analysis suggested that the lower exercise capacity documented in patients with OSA is not obvious in moderate to severe OSA, whereas subgroup analysis conducted with aggregate meta-analysis has revealed that only severe OSA is associated with a lower peak VO2 as compared to that in controls [14]. 

Positive airway pressure (PAP) is the gold-standard treatment option for symptomatic OSA patients, but its use is limited by poor PAP adherence [1]. The use of PAP might prevent subsequent cardiovascular events, which has been only demonstrated in observational studies, not in randomized controlled trials [15]. There is still no significant association of PAP treatment with risk reduction in composite cardiovascular events or all-cause and cardiovascular mortality in patients with OSA and CVD [15]. Moreover, the results from the existing literature regarding the effect of PAP treatment on CPET-measured exercise capacity parameters in OSA patients are inconsistent and controversial [16].

The objective of this study is to investigate the impact of adherence to PAP therapy on CPET performance in patients with concomitant OSA and CVD. We hypothesized that adherence to PAP therapy is associated with an improvement in CRF in patients with OSA and comorbid CVD. Therefore, we compared the changes in CPET parameters before and after PAP treatment between patients who were adherent to PAP and patients who were not adherent to PAP.

## 2. Materials and Methods

### 2.1. Study Population and Protocol 

Between February 2021 and May 2022, we prospectively enrolled consecutive newly diagnosed OSA patients requiring PAP treatment who were followed regularly for CVD by a cardiology specialist in Daejeon St. Mary Hospital at the Catholic University of Korea. OSA diagnosis was made by full-night polysomnography. According to the American Academy of Sleep Medicine, the study criterion for OSA was an AHI ≥ 5 events/h with symptoms or AHI ≥ 15 events/h regardless of the presence of symptoms [17]. Besides a history of CVD, including hypertension, coronary disease, or heart failure (HF), the other inclusion criteria were as follows: aged ≥19 years and clinically stable for 3 months on optimized treatment, including medication or intervention. The exclusion criteria were pregnancy, prior use of PAP, significant heart, lung, or musculoskeletal diseases that would impede CPET, and neuropsychiatric diseases that would prevent the patients from understanding and following the study protocol. At the time of study enrollment before initiation of the PAP therapy, all subjects underwent comprehensive clinical investigations, which included an in-depth physical examination and recording of symptoms, medical and surgical histories, current medication, and comorbidities, full-night polysomnography, echocardiography, and CPET. After 6 to 12 months of PAP treatment, the CPET performance was re-assessed in all subjects. The study was conducted in accordance with the Declaration of Helsinki, and the protocol was approved by the Institutional Review Board (DC20OISI0095). All participants provided written informed consent. 

### 2.2. Echocardiography 

Echocardiography was performed using commercially available equipment (Vivid E9, GE Medical Systems, Horten, Norway or Phillips EPIQ, Philips Medical Systems, Andover, MA, USA). Two-dimensional images were obtained from the standard parasternal and apical windows. The modified biplane Simpson’s method was used to measure left ventricular end-diastolic and end-systolic volumes, from which the left ventricular ejection fraction (LVEF) was derived. Tissue Doppler e’ was measured in the septum and lateral wall, and the average value was used to obtain E/e’. Echocardiography was performed to confirm the stable status of patients at the time of study enrollment.

### 2.3. Polysomnography

Full-night polysomnography was performed under the supervision of trained professionals using a digital system (Nox A1^®^ PSG system, Nox Medical, Reykjavik, Iceland) in the sleep laboratory during the patient’s habitual sleep time. We monitored an electroencephalogram (three channels), electrooculogram (left and right eyes), electromyogram (submental and anterior tibialis muscles), electrocardiogram, oronasal airflow (pressure transducer and a thermistor), thoracoabdominal excursions (inductance plethysmography), snoring, body position sensor, oxyhemoglobin saturation (pulse oximetry), and pulse rate. Recordings were manually scored by an experienced sleep technician and reviewed by a sleep medicine physician. Apnea was defined as a ≥90% decrease in airflow for a minimum of 10 s [17]. Hypopnea was defined as a ≥30% reduction in airflow lasting ≥10 s, associated with a ≥4% desaturation [17].

### 2.4. Positive Airway Pressure Therapy and Adherence 

Before initiating PAP treatment, an upper airway examination by an ear, nose, and throat specialist was performed. Auto-adjusting PAP (APAP) was used for PAP therapy. Participants treated with APAP were started on a 4–20 cm H_2_O pressure, which could be adjusted as clinically indicated. There were no restrictions regarding the type of mask interface, and participants were allowed to change the size and model of their mask interface throughout the study. The adherence data were obtained from device downloads and the average use in hours per day across the treatment period was recorded. According to the US Centers for Medicare and Medicaid Services criteria [18], adherence was defined as usage of the device for at least 4 h per night on 70% of the nights during a consecutive 30-day period in the first 90 days of therapy.

### 2.5. Cardiopulmonary Exercise Testing 

All patients underwent CPET (Ultima CPX^TM^, MGC Diagnostics, Saint Paul, MN, USA) according to the MOD-Bruce protocol using a treadmill (GE T-2000, CardioSoft diagnostic software version 5.20, Marquette ECG analysis program, GE Medical Systems IT, Inc., Milwaukee, WI, USA). Heart rate was continuously monitored by electrocardiography, and blood pressure was measured at rest and every 2 min thereafter. Breath-by-breath oxygen consumption (VO2), carbon dioxide production (VCO2), and minute ventilation (VE) were collected using a ventilatory expired gas analysis system. The test termination criteria were the patient’s exhaustion due to symptoms, ventricular arrhythmia, ST segment depression ≥ 2.0 mm, and a drop in systolic blood pressure ≥ 20 mmHg. Peak VO2 was defined as the highest average VO2 during the peak exercise. Percentage values of the predicted peak VO2 were calculated using the Wasserman formula [19]. The VE/VCO2 slope was calculated to estimate the ventilatory response to exercise by software.

### 2.6. Statistical Analysis 

Continuous variables were expressed as mean ± standard deviation or median (interquartile range), and they were analyzed by the *t*-test or Wilcoxon rank-sum test. Categorical variables were presented as frequencies and percentages, and they were analyzed by the Chi-square test or Fisher exact test. All statistical analyses were performed with SAS 9.4 (SAS Institute, Cary, NC, USA). All tests were two-tailed, and *p* < 0.05 was considered significant.

## 3. Results

### 3.1. Study Population and Baseline Characteristics

**Figure 1:**
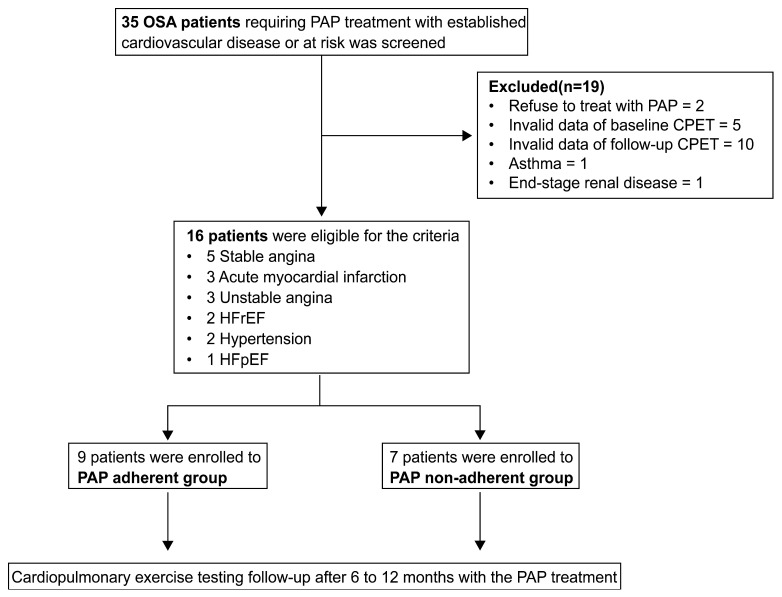
Study population.

A flow chart of the study group is shown in Figure 1. From among 35 OSA patients who were prospectively enrolled, 19 patients were excluded. Two patients did not agree to undergo treatment with PAP, five patients had invalid data of baseline CPET, ten patients had invalid data of follow-up CPET, one patient had asthma, and one patient had end-stage renal disease and was on hemodialysis. A total of 16 OSA patients (12 males) with a median age of 67.0 [60.5–70.0] years and a BMI of 26.2 [24.5–28.6] kg/m^2^ were included in the final analysis. The median AHI was 23.2 [16.9–39.7] events/h. Concomitant CVD included coronary disease in 11 patients, heart failure in three patients, and two well-controlled arterial hypertensive patients without other CVDs. Patients were classified into the adherent (*n* = 9) and non-adherent (*n* = 7) groups with respect to PAP adherence. Demographic and clinical characteristics of the study population did not significantly differ between the two groups (Table 1). 

Among the 35 patients with OSA, 16 patients were finally eligible for the study. A total of 16 OSA patients were classified into the adherent (*n* = 9) and non-adherent (*n* = 7) groups with respect to PAP adherence. 

OSA, obstructive sleep apnea; PAP, positive airway pressure; CPET, cardiopulmonary exercise testing; HFrEF, heart failure with reduced ejection fraction; HFpEF, heart failure with preserved ejection fraction. 

### 3.2. Echocardiographic and Polysomnographic Parameters 

On comparing the baseline echocardiographic parameters, the echocardiographic parameters did not differ between the two groups (Table 2). Baseline sleep-related questionnaires and polysomnographic parameters are shown in Table 3. No significant differences were found in sleep-related questionnaires between the two groups. The arousal index was significantly higher in the PAP-adherent group compared to the PAP-non-adherent group (33.7 [33.2–39.0] events/h vs. 11.6 [5.2–16.9] events/h, *p* = 0.018). Baseline AHI was higher in the PAP-adherent group compared to the PAP-non-adherent group (35.1 [24.0–40.5] events/h vs. 16.3 [11.3–21.9], *p* = 0.042). 

### 3.3. Changes in Cardiopulmonary Exercise Testing Parameters 

The changes in CPET parameters before and after PAP treatment between patients who were adherent to PAP and patients who were not adherent to PAP are shown in Table 4. After 6 to 12 months of PAP treatment, the PAP-adherent group showed a greater increase in peak VO2 than the PAP-non-adherent group without any statistical significance (Δ = 3.0 ± 4.5 mL/kg/min vs. 1.1 ± 8.8 mL/kg/min, *p* = 0.581). The decrease in VE/VCO2 was significantly greater in the PAP-adherent group compared to the PAP-non-adherent group (Δ = −8.6 ± 3.9 vs. −1.7 ± 7.3, *p* = 0.030).

## 4. Discussion

We used the real-world data of prospectively enrolled consecutive patients with concomitant OSA and CVD to demonstrate the impact of PAP therapy on cardiorespiratory efficiency and ventilatory drive during exercise. The major finding of this study is that adherence to PAP therapy for OSA in patients with CVD is associated with an improvement in exercise performance, especially in the VE/VCO2 slope, as an index of the ventilatory response to exercise. 

CPET has proven to be a valuable tool for evaluating exercise capacity and predicting outcomes in HF and other cardiopulmonary conditions [9]. The peak VO2 is the most widely used CPET parameter, which has consistently demonstrated prognostic significance in patients with HF [20,21,22]. However, the critical disadvantage of peak VO2 is the need for maximal exercise [23]. Moreover, peak VO2 might be underestimated due to reduced patient motivation as well as premature termination of the test by the examiner [23,24]. For these reasons, new CPET parameters have been demonstrated to have a clinical prognostic value. Among the variables, including the peak VO2, percentage of predicted peak VO2, VE/VCO2 slope, oxygen uptake efficiency slope, and peak oxygen uptake efficiency, the VE/VCO2 slope has been demonstrated to be a strong prognostic factor associated with cardiovascular events (HR 11.14) [25]. Reindl I et al. demonstrated that VE/VCO2 was correlated with exercise intolerance and cardiac output [26]. Recently, several studies have also emphasized that the VE/VCO2 slope, the ratio of minute ventilation over CO2 output, is an excellent prognostic parameter, and it improves the risk stratification of HF patients [27,28,29]. 

In the current study, the PAP-adherent group showed a greater increase in peak VO2 than the PAP-non-adherent group, without any statistical significance. The VE/VCO2 slope was significantly decreased in the PAP-adherent group compared to the PAP-non-adherent group. These results indicate the critical role of exaggerated ventilatory control and augmented chemosensitivity in the pathogenesis of OSA in patients with CVD [2,30]. Because of an overshoot in ventilation in response to a small amount of breathing reduction during sleep, augmented chemosensitivity to CO2 promotes ventilatory instability [27]. Patients with HF exhibited enhanced hypercapnic and hypoxic ventilatory responses [27], and these ventilatory responses were obtained during rest and were closely associated with the ventilatory response to exercise, determined by the VE/VCO2 slope [31]. Bittencourt et al. reported that the slope of VE/VCO2 obtained during exercise was correlated with the severity of OSA, suggesting that an elevated ventilatory response should increase suspicion of the presence of severe OSA in patients with HF [32].

There has been disagreement over how PAP therapy affects the CPET-measured exercise tolerance in patients with OSA [16]. Quadri et al. aimed to evaluate the impact of continuous PAP (CPAP) on exercise performance and cardiovascular autonomic anomalies in patients with OSA who did not alter their lifestyle or weight while receiving therapy [33]. Although their BMIs did not change, two months of CPAP treatment improved the peak VO2 and physical exercise tolerance [33]. Another study also showed an improvement in CRF in patients with OSA after two months of CPAP treatment [34]. On the other hand, no noticeable difference in the peak VO2 was reported throughout the 3-month PAP therapy period [35]. The duration of PAP treatment varied in these studies, making it difficult to distinguish between the short- and long-term effects. In addition, a wide range of other health issues in OSA patients have added to the controversy related to this subject.

Although the effects of OSA per se on the cardiovascular response to exercise remain unknown, the associated cardiovascular comorbidities may lower the cardiopulmonary exercise capacity. Therefore, PAP treatment is likely to be effective in improving the CPET performance in patients with concomitant OSA and CVD. Among the CPET parameters, VE/VCO2 might be useful to evaluate the effect of PAP treatment that reflects the pathophysiology of OSA in CVD patients. This preliminary study could provide evidence for the potential utility of CPET in the clinical management of these patients, but its main limitation was a small sample size and a relatively short follow-up duration. However, we compared the difference between the baseline and follow-up CPET variables after stabilizing patients’ treatment, including medication or intervention, which could minimize bias in each patient by controlling unmeasurable confounding factors. Another limitation was a lack of follow-up polysomnography and echocardiography after PAP treatment. Further research is needed to overcome these limitations. 

## 5. Conclusions

Adherence to PAP therapy for OSA in patients with CVD is associated with an improvement in the CPET performance, especially in the VE/VCO2 slope, as an index of the ventilatory response to exercise. This suggests that greater emphasis should be placed on diagnosing and effectively treating OSA with PAP in patients with CVD. Screening for sleep apnea in patients with CVD may be warranted, and strategies to optimize adherence to PAP in these patients are beneficial. Further evidence is needed to elucidate whether CPET could be routinely used to monitor treatment responses of OSA to PAP therapy in patients with CVD.

## Figures and Tables

**Table 1:** Demographic and clinical characteristics according to PAP adherence (*n* = 16).

	PAP-Non-Adherent (*n* = 7)	PAP-Adherent (*n* = 9)	*p* Value
Age, years	68 (61–71)	66 (60–69)	0.381
Male, *n* (%)	4 (57.1)	8 (88.9)	0.262
BMI, kg/m^2^	26.1 (23.9–27.7)	26.6 (24.7–29.4)	0.604
Hypertension, *n* (%)	4 (57.1)	8 (88.9)	0.262
Diabetes, *n* (%)	2 (28.6)	3 (33.3)	>0.999
Dyslipidemia, *n* (%)	7 (100.0)	7 (77.8)	0.475
COPD, *n* (%)	1 (14.3)	0 (0.0)	0.438
Current smoking, *n* (%)	1 (14.3)	1 (11.1)	>0.999
Medication, *n* (%)			
ACE inhibitor or ARB	1 (14.3)	5 (55.6)	0.145
Beta-blocker	3 (42.9)	6 (66.7)	0.615
Diuretics	3 (42.9)	1 (11.1)	0.262
Platelet antiaggregant	6 (85.7)	7 (77.8)	>0.999
Lipid-lowering agent	7 (100.0)	7 (77.8)	0.810
Laboratory data			
FBS, mg/dL	109 (101–138)	107 (96–121)	0.834
Hemoglobin, g/dL	13.4 ± 1.5	14.1 ± 1.4	0.378
Creatinine, mg/dL	0.9 ± 0.2	0.8 ± 0.2	0.857
eGFR, mL/min/1.73 m^2^	86.7 ± 20.8	98.5 ± 20.9	0.279
TC, mg/dL	165.7 ± 47.1	148.0 ± 47.1	0.480
Triglyceride, mg/dL	140.1 ± 56.9	155.0 ± 51.9	0.606
HDL-C, mg/dL	60.0 ± 10.7	47.9 ± 11.9	0.069
LDL-C, mg/dL	94.2 ± 43.1	82.7 ± 41.0	0.606
hs CRP, mg/dL	0.09 (0.07–0.10)	0.16 (0.05–0.90)	0.434
HbA1c, %	6.1 (5.7–7.0)	6.2 (5.4–6.3)	0.574

BMI, body mass index; COPD, chronic obstructive pulmonary disease; ACE inhibitor, angiotensin-converting enzyme inhibitor; ARB, angiotensin receptor inhibitor; FBS, fasting blood glucose; eGFR, estimated glomerular filtration rate; TC, total cholesterol; HDL-C, high-density lipoprotein cholesterol; LDL-C, low-density lipoprotein cholesterol; CRP, C-reactive protein; HbA1c, glycosylated hemoglobin. Values are presented as the mean ± SD or median (interquartile range).

**Table 2:** Echocardiographic parameters according to PAP adherence (*n* = 16).

	PAP-Non-Adherent (*n* = 7)	PAP-Adherent (*n* = 9)	*p* Value
LVESV, mL/m^2^	42.4 ± 25.3	50.4 ± 29.7	0.444
LVEDV, mL/m^2^	75.6 ± 22.6	89.4 ± 30.3	0.462
LV EF, %	59.5 ± 17.9	51.4 ± 14.2	0.386
E, m/s	52.3 ± 12.5	71.0 ± 18.9	0.064
A, m/s	66.4 ± 12.5	76.5 ± 43.4	0.593
E/A	0.8 (0.8–0.8)	0.7 (0.5–0.8)	0.331
Average E/e’	6.5 (5.7–7.0)	10.7 (6.6–14.2)	0.099

LV, left ventricular; LVEDV, LV end-diastolic volume; LVESV, LV end-systolic volume; EF, left ventricular ejection fraction; E, early diastolic transmitral flow velocity; A, late diastolic transmitral flow velocity; e’, early diastolic relaxation velocity at septal mitral annular position. Values are presented as the mean ± SD or median (interquartile range).

**Table 3:** Sleep-related questionnaires and polysomnographic parameters according to PAP adherence (*n* = 16).

	PAP-Non-Adherent (*n* = 7)	PAP-Adherent (*n* = 9)	*p* Value
Sleep-related questionnaires			
PSQI	11.1 ± 6.9	14.6 ± 6.2	0.318
ISI	2.0 (1.0–7.0)	6.0 (3.0–6.0)	0.407
SSS	2.0 (2.0–2.0)	3.0 (2.0–3.0)	0.253
ESS	3.0 (1.0–6.0)	6.0 (3.0–7.0)	0.094
BQ	1.9 ± 1.7	2.4 ± 1.6	0.486
STOP-BANG	2.9 ± 1.6	4.0 ± 1.3	0.137
BDI	11.0 (9.0–22.0)	13.0 (12.0–14.0)	0.501
BAI	7.0 (6.0–10.0)	5.0 (4.0–8.0)	0.433
Polysomnography			
TST, min	396.6 ± 38.8	396.7 ± 75.7	0.999
SE, %	89.1 (86.0–91.0)	89.8 (84.6–94.0)	0.958
SL, min	9.6 (4.9–22.5)	12.8 (8.4–25.5)	0.410
N1, % TST	7.5 ± 2.1	8.4 ± 4.2	0.627
N2, % TST	46.5 ± 12.0	56.9 ± 12.3	0.111
N3, % TST	28.9 ± 9.0	19.2 ± 9.1	0.053
REM, % TST	15.8 ± 4.7	15.5 ± 5.1	0.912
AI, events/h	11.6 (5.2–16.9)	33.7 (33.2–39.0)	0.018 *
AHI, events/h	16.3 (11.3–21.9)	35.1 (24.0–40.5)	0.042 *
OAI, events/h	2.1 (0.7–5.2)	11.1 (4.2–30.4)	0.111
MAI, events/h	0 (0–0.3)	0.8 (0.6–1.0)	0.148
CAI, events/h	0 (0–1.4)	0.9 (0.2–1.3)	0.427
RDI, events/h	17.8 (11.3–21.5)	36.6 (25.8–41.0)	0.042 *
Lowest SaO_2_, %	83.7 ± 6.7	80.9 ± 6.8	0.419
SaO_2_ < 90%, % TST	1.1 (0.1–1.1)	6.7 (1.5–23.5)	0.187
ODI, %	16.1 (12.5–20.7)	33.0 (23.2–39.7)	0.042 *

PSQI, Pittsburgh sleep quality index; ISI, insomnia severity index; SSS, Stanford sleepiness scale; ESS, Epworth sleepiness scale; BQ, Berlin questionnaire; BDI, Beck depression inventory; BAI, Beck anxiety inventory; TST, total sleep time; SE, sleep efficiency; SL, sleep latency; REM, rapid eye movement; AI, arousal index; AHI, apnea–hypopnea index; OAI, obstructive apnea index; MAI, mixed apnea index; CAI, central apnea index; RDI, respiratory distress index; ODI, oxygen desaturation index. Values are presented as the mean ± SD or median (interquartile range). * *p* value < 0.05.

**Table 4:** Changes in cardiopulmonary exercise testing parameters with respect to PAP adherence.

	PAP-Non-Adherent (*n* = 7)			PAP-Adherent (*n* = 9)			
	Baseline	Follow Up	Δ Change	Baseline	Follow Up	Δ Change	*p* Value Δ Group
Resting SBP, mmHg	138.4 ± 23.8	129.7 ± 19.5	−8.7 ± 21.6	135.0 ± 27.9	125.7 ± 18.9	−9.3 ± 13.9	0.945
Resting DBP, mmHg	84.7 ± 14.2	79.1 ± 15.4	−5.6 ± 5.1	82.6 ± 15.4	82.2 ± 15.3	−0.3 ± 8.2	0.161
Resting HR, bpm	86.6 ± 10.0	77.3 ± 16.2	−9.3 ± 12.2	80.0 ± 19.1v	75.4 ± 11.2	−4.6 ± 11.9	0.448
Peak SBP, mmHg	178.4 ± 39.0	165.6 ± 26.8	−12.9 ± 30.8	178.8 ± 30.0	168.6 ± 28.9	−10.2 ± 26.7	0.857
Peak DBP, mmHg	77.6 ± 18.4	77.0 ± 20.8	−0.6 ± 28.1	85.7 ± 20.2	84.9 ± 15.8	−0.8 ± 16.0	0.985
Peak HR, bpm	141.1 ± 13.4	126.6 ± 18.9	−14.6 ± 14.8	135.9 ± 22.6	134.8 ± 21.5	−1.1 ± 14.2	0.086
Peak VO2, mL/kg/min	22.1 ± 6.4	23.2 ± 10.9	1.1 ± 8.8	21.4 ± 7.0	24.4 ± 6.6	3.0 ± 4.5	0.581
	19.6 (17.7–27.7)	23.2 (11.5–34.6)	3.2 (−4.5–6.9)	21.7 (16.9–25.9)	25.1 (18.7–27.2)	2.8 (0.7–5.5)	0.832
Peak VO2, %	79.5 ± 6.2	75.7 ± 20.8	−7.6 ± 39.7	66.2 ± 19.6	80.0 ± 33.5	13.8 ± 19.8	0.687
VE/VO2 slope	34.3 ± 8.2	26.9 ± 4.7	−7.4 ± 7.5	37.9 ± 15.4	33.7 ± 15.9	−4.2 ± 10.5	0.505
VE/VCO2 slope	30.0 ± 2.9	28.3 ± 6.2	−1.7 ± 7.3	30.9 ± 5.1	22.3 ± 7.3	−8.6 ± 3.9	0.030 *
	30.0 (29.0–31.0)	29.0 (24.0–31.0)	−2.0 (−6.0–6.0)	29.0 (29.0–34.0)	21.0 (17.0–25.0)	−9.0 (−12.0–−6.0)	0.038 *
METs	7.3 ± 3.2	7.8 ± 3.4	0.4 ± 2.0	7.3 ± 2.9	7.8 ± 2.4	0.5 ± 1.2	0.959

SBP, systolic blood pressure; DBP, diastolic blood pressure; HR, heart rate; METs, metabolic equivalents. Values are presented as the mean ± SD or median (interquartile range). Δ Change is the difference from baseline to follow-up. *p* value Δ group designates the difference between the PAP-non-adherent group and the PAP-adherent group in the change between baseline and follow-up values. * *p* value < 0.05.

## Data Availability

Data is available upon reasonable request from the corresponding author.
